# Tranylcypromine: a promising repurposed drug against Leishmaniases

**DOI:** 10.3389/fmicb.2025.1756231

**Published:** 2026-01-12

**Authors:** Rafeh Oualha, Yosser Zina Abdelkrim, Khadija Essafi-Benkhadir, Ikram Guizani, Emna Harigua-Souiai

**Affiliations:** Laboratory of Molecular Epidemiology and Experimental Pathology-LR16IPT04, Institut Pasteur de Tunis, Université de Tunis El Manar, Tunis, Tunisia

**Keywords:** drug discovery, drug repurposing, *in vitro* validation, Leishmaniases, tranylcypromine

## Abstract

Leishmaniases remain a major global health challenge due to limited and often toxic therapeutic options. Recent advances in artificial intelligence have enabled the identification of novel antileishmanial candidates through drug repurposing approaches. Among these, our team has previously predicted in *silico* the monoamine oxidase inhibitor Tranylcypromine as a potential drug candidate against Leishmaniases. In this study, we provided the first experimental evidence supporting its efficacy against *Leishmania major* using both promastigote and intracellular amastigote models. Tranylcypromine markedly decreased parasite viability in a concentration-dependent manner, displaying IC_50_ values of 83.6 and 31.6 μg/ml against promastigotes and amastigotes, respectively. Mechanistic investigations revealed that at concentrations ranging from 50 to 200 μg/ml, it induced an apoptotic death of *L. major* promastigotes leading to necrosis at higher concentrations. Viability and cytotoxicity assays on THP-1-derived macrophages highlighted that the compound, at the selected concentrations, was safe and did not induce a toxic effect on host cells with CC_50_ values exceeding 280 μg/ml. Altogether, these findings revealed Tranylcypromine as a selective and promising antileishmanial drug candidate, supporting the relevance of AI-assisted drug repurposing strategies to accelerate drug discovery of safe and affordable therapies for neglected tropical diseases.

## 1 Introduction

Leishmaniases are a group of neglected tropical diseases caused by intracellular protozoan parasites of the genus *Leishmania*. They represent a major global health challenge, with an estimated 700,000 to 1 million new cases and 26,000 to 65,000 deaths annually, according to the World Health Organization (WHO). Despite their burden, these diseases remain largely neglected. Currently, there is no vaccine, and the first-line treatments rely on pentavalent antimonials (e.g., Glucantime®, Pentostam®), which are associated with high toxicity (Altamura et al., 2022; Masne et al., 2024). Second-line therapies, mainly repurposed drugs such as Amphotericin B, Pentamidine, Miltefosine, and Paromomycin, are limited by severe adverse effects, high costs, and the emergence of drug resistance (Croft et al., 2006; Altamura et al., 2022; Masne et al., 2024; Zhang et al., 2025). The urgent need for new, safer, and affordable therapies has renewed interest in drug repurposing, an approach that seeks new applications for approved drugs with established safety profiles.

Drug repurposing relies on the concept of identifying new therapeutic applications for approved drugs, and represents a promising strategy for drug discovery against neglected tropical diseases (Zhan et al., 2022; Mentis et al., 2024). It significantly reduces both the time and cost of the development process compared to novel drug discovery through shortening the path from early steps of discovery to the clinical trials. Pre-existing pharmacokinetic and safety data provide an asset for low-withdrawal risk throughout the pipeline. Drug repurposing can be classified into two key categories: “on-target” and “off-target” approaches. On-target repurposing involves using drugs for new applications based on knowledge of their action against a molecular target. This includes disease-centric approaches, which exploit shared biological pathways between diseases to find new uses for existing drugs, and target-centric approaches, which focus on drugs acting on specific validated molecular targets implicated in different diseases (Harigua-Souiai et al., 2018; Pushpakom et al., 2019; Nossier et al., 2025). In contrast, “off-target” repurposing identifies novel therapeutic effects arising from a drug interacting with previously unrecognized targets, expanding potential applications beyond the drug’s initial mechanism (Pushpakom et al., 2019; Nossier et al., 2025). Together, these strategies leverage existing pharmacological and clinical data, supported by computational and experimental tools, to accelerate drug discovery. Given current challenges in drug innovation, advanced computational techniques based on artificial intelligence (AI), including machine learning and deep learning, are revolutionizing drug discovery by enabling efficient analysis of large-scale biological and chemical data (Harigua-Souiai et al., 2024; Masmoudi et al., 2025). These methods accelerate drug repurposing through generative modeling, binding site prediction, and similarity-based screening of existing compounds (Harigua-Souiai et al., 2015; Pushpakom et al., 2019; Moingeon, 2021; Wan et al., 2025). In the context of leishmaniases, such computational strategies have supported the identification of promising repurposed candidates. Building on these principles and methodologies, numerous molecules from diverse therapeutic classes have been successfully repurposed for leishmaniases (Andrade-Neto et al., 2018; Lye et al., 2024; Oualha et al., 2024).

The diversity of compound classes include antifungal, anticancer, antibiotic, anti-depressive and antihypertensive agents, among others, highlighting the broad applicability and success of drug repurposing across various pharmacological categories. Many repurposed drugs have modes of action that remain poorly understood, reflecting both the complexity of parasitic diseases and existing gaps in mechanistic understanding.

In a previous study using machine learning (ML), we developed a drug repurposing strategy to identify FDA-approved drugs with potential anti-*Leishmania* activity (Harigua-Souiai et al., 2022). A dataset of 65,057 compounds tested against *Leishmania major* was used to train and optimize ML models. Random Forest (RF) and Support Vector Machine (SVM) algorithms showed the best performances and were used to assess the anti-*Leishmania* potential effects of FDA-approved drugs. This led to the prediction of drug candidates against leishmaniases (Harigua-Souiai et al., 2022), among which 10 were experimentally tested by our group, validating three novel anti-*Leishmania* therapeutic agents (Oualha et al., 2024). Within the predicted anti-*Leishmania* candidates that did not undergo the experimental validation, tranylcypromine, an FDA-approved monoamine oxidase inhibitor (MAOI) primarily used to treat major depressive disorder, emerged as a promising hit with a confidence score of 80% (Harigua-Souiai et al., 2022). To our knowledge, it has never been experimentally tested against leishmaniases. Tranylcypromine acts by irreversibly inhibiting monoamine oxidase enzymes, leading to increased levels of monoamine neurotransmitters. Beyond its psychiatric indications, its well-characterized pharmacological profile, structural adaptability, and favourable safety record make it an attractive molecule for drug repurposing. Tranylcypromine hydrochloride was previously identified among FDA-approved drugs that inhibit the proteolytic activity of SARS-CoV-2 3CLpro (Chiou et al., 2021). These features, together with its potential to modulate multiple biochemical pathways, support its investigation as a potential antileishmanial agent.

In the present study, our objective was to build on the predictive outcomes from the study of Harigua-Souiai et al., 2022, providing experimental validation of the effects of tranylcypromine on *Leishmania* parasites. We provide here the first experimental evidence confirming the predicted anti-*Leishmania* activity of tranylcypromine. We assessed its efficacy against both extracellular promastigotes and intracellular amastigotes of *L. major*, and evaluated its mechanism of action through apoptosis and necrosis assays. Cytotoxicity was also evaluated on THP-1–derived macrophages to determine its selectivity toward the parasite.

## 2 Materials and methods

### 2.1 Parasites and cell culture

The *L. major* Empa-12 strain (MHOM/TN/2012/Empa-12), originally isolated from a patient with zoonotic cutaneous leishmaniasis (Oualha et al., 2019, 2024), was used in this study. Parasites were cultured at 22 °C in RPMI-1640/Glutamax medium (Gibco BRL, Germany) supplemented with penicillin (100 U/ml), streptomycin (100 μg/ml) and 10% heat-inactivated fetal bovine serum (FBS) (Gibco, Germany).

The human myelomonocytic cell line THP-1 was obtained from the American Type Culture Collection (ATCC, TIB-202). Cells were cultured in RPMI 1640/Glutamax-I medium (Gibco BRL, Germany) supplemented with streptomycin (100 μg/ml) and penicillin (100 U/ml) and 10% heat-inactivated fetal bovine serum (FBS) (Gibco, Germany) at 37 °C, 5% CO_2_. To induce differentiation into macrophage-like cells, THP-1 cells were treated with 25 ng/ml phorbol 12-myristate 13-acetate (PMA) (Sigma, St. Louis, MO, USA). PMA-treated THP-1 cells were maintained under these conditions for 24 h, followed by a 24-h resting period after PMA removal.

### 2.2 Drug preparation and treatment

Tranylcypromine hydrochloride (Ref. HY-17447A) was purchased from MedChemExpress. Stock solutions were prepared in DMSO and stored at −30 °C. All subsequent dilutions were freshly prepared in RPMI-1640 medium on the day of experiment. Mock-treated promastigotes and THP-1 cells in complete medium containing 0.5% DMSO (without drug) served as the negative control (Mock). For all experiments, both compounds and controls were applied at a final DMSO concentration of 0.5% per well.

### 2.3 Anti-promastigotes assay

The anti-promastigote activity of Tranylcypromine was assessed by MTT assay. Stationary-phase promastigotes of *L. major* strain (5 × 10^5^ parasites/well) were incubated in 96-well plates with increasing concentrations of Tranylcypromine (0, 4.68, 6.25, 9.37, 12.5, 18.75, 25, 37.5, 50, 75, 100, 150, 200, 300 and 400 μg/ml) for 24 h at 26 °C. After incubation, MTT (5 mg/ml) was added and formazan crystals were solubilized in DMSO. Absorbance was measured at 570 nm, and IC_50_ values were calculated using a nonlinear regression model of the logarithm of compound concentrations (GraphPad Prism 9). The viability of Empa-12 promastigotes was expressed as the percentage of viable parasites in Tranylcypromine-treated wells relative to mock-treated controls. IC_50_ values were reported as the mean ± SD of three independent experiments performed in duplicate.

### 2.4 Evaluation of parasite morphology following compound exposure

To assess the effect of the compound on parasite morphology, promastigotes of the *L. major* strain (5 × 10^5^ parasites/well) were incubated in 96-well plates with increasing concentrations of Tranylcypromine (0, 25, 50, 75, 100, 150, 200 and 400 μg/ml) at 26 °C. After for 24 h of incubation, parasite suspensions were placed on glass slides, fixed, and stained using May-Grunwald-Giemsa method with the RAL 555 Kit (RAL DIAGNOSTICS, France), following the manufacturer’s instructions. Morphological features were examined microscopically at 100x magnification and images were captured under oil immersion using the front camera of smartphone, with a 48-megapixel (MP) resolution. The smartphone was mounted on a NexYZ 3-Axis Universal Smartphone Adapter (Celestron) to ensure image stability and consistent focusing during acquisition. Image acquisition parameters were identical for all samples, and multiple fields were imaged per condition. This in-house smartphone system has been validated for morphological analysis and parasites detection using computer vision systems, trained on a curated dataset of 180 images, that was collected by our group and was made publicly available (Harigua et al., 2025).

### 2.5 Apoptosis and necrosis assay

Apoptotic and necrotic responses were assessed using the RealTime-Glo™ Annexin V Apoptosis and Necrosis Assay (Promega), following the manufacturer’s protocol. Briefly, stationary-phase of *Leishmania* promastigotes (5 × 10^5^ cells per well) were exposed to various concentrations of Tranylcypromine (0, 4.68, 6.25, 9.37, 12.5, 18.75, 25, 37.5, 50, 75, 100, 150, 200, 300 and 400 μg/ml) in 96-well plates. After reagent addition, luminescence signal was used to quantify apoptosis, while fluorescence (excitation 485 nm/emission 510 nm) indicated the loss of plasma membrane integrity and necrosis. Signal measurements were recorded after 24 h of incubation at room temperature using a microplate reader. Miltefosine (1.25 μg/ml) was used as a positive control for apoptosis-like cell death based on it is well-established activity as antileishmanial drug that induces a programmed cell death in *Leishmania* parasites (Khademvatan et al., 2011). The detergent Triton X-100 (1%) was used as positive necrosis control due to its rapid disruption of plasma membrane integrity. Data are presented as mean ± SD from at least three independent experiments. Student’s t-tests were used to assess the significance of differences between means, with *p*-values < 0.05 indicating statistically significant differences.

### 2.6 Cell viability assay (MTT)

To evaluate the effect of Tranylcypromine on the viability of THP-1-derived macrophages, 5 × 10^4^ differentiated THP-1 macrophages were treated with increasing concentrations of Tranylcypromine (0, 25, 37.5, 50, 75, 100, 150, 200, 300, and 400 μg/ml). After 24 h of incubation, 25 μl of MTT solution (5 mg/ml) was added to each well and further incubated at 37 °C with 5% CO_2_ for 4 h to assess the effect of Tranylcypromine on cell viability. The viability of THP-1-derived macrophages was expressed as the percentage of viable cells in Tranylcypromine-treated wells relative to mock-treated controls. Log-transformed Tranylcypromine concentrations were plotted against response percentages (% viability), and nonlinear regression analysis was performed to determine CC_50_ (the concentration of compound that reduces cell viability by 50%) values using GraphPad Prism version 9.0.1. CC_50_ values were reported as the mean ± SD of -three independent experiments performed in duplicate.

### 2.7 Cell cytotoxicity assay (LDH)

The cytotoxicity of Tranylcypromine toward THP-1-derived macrophages was assessed using the LDH assay, which quantifies lactate dehydrogenase (LDH) released into the culture medium as a result of plasma membrane damage. Human monocytic THP-1 cells were cultured and differentiated into macrophages as described previously, then incubated with mock (0.5% DMSO) or Tranylcypromine at different concentrations (0, 25, 37.5, 50, 75, 100, 150, 200, 300, and 400 μg/ml). Cells treated with 1% Triton X-100 served as a positive control for cell lysis. After 24 h of incubation, culture supernatants were collected and incubated for 30 min at 37 °C with the reaction substrate provided in the Cytotoxicity Detection Kit LDH (Roche). Extracellular LDH activity was measured spectrophotometrically, and cytotoxicity was expressed as the percentage of cytotoxicity relative to the positive control (100% cytotoxicity induced by 1% Triton X-100). Dose–response curves were generated by plotting the logarithm of Tranylcypromine concentrations against the percentage of cytotoxicity. CC_50_ values (the concentration of compound that induces 50% cytotoxicity) were determined by nonlinear regression analysis of log-transformed concentrations versus response percentages using GraphPad Prism version 9.0.1. CC_50_ values were reported as the mean ± SD of three independent experiments performed in duplicate.

### 2.8 Anti-amastigotes assay

The antileishmanial activity of Tranylcypromine against intracellular amastigotes was assessed by infecting THP-1-derived macrophages with stationary-phase *L.major*. To this end, (5 × 10^4^) cells per well were seeded in each 8-well Labtek slides (Thermo Scientific). Adherent macrophages were infected with (Empa-12) promastigotes at a parasite-to-cell ratio of 10:1 for 24 h at 37 °C. After removal of extracellular parasites, infected macrophages were incubated for 24 h with Tranylcypromine at concentrations of 0, 9.37, 18.75, 25, 37.5, 50, 75, and 100 μg/ml. Cells were then fixed, stained with RAL 555, and examined microscopically at 100x magnification. For each condition, at least 100 macrophages were analyzed to determine the percentage of infected cells and the mean number of intracellular amastigotes per 100 macrophages. The percentage of inhibition was expressed as the percentage of total number of internalized parasites within Tranylcypromine-treated cells relative to the total number of internalized parasites within control. IC_50_ values were determined from log-transformed dose–response curves by nonlinear regression analysis using GraphPad Prism v9.0.1. IC_50_ values were reported as the mean ± SD of three independent experiments.

## 3 Results

### 3.1 Tranylcypromine reduced promastigote viability in a dose-dependent manner

**Figure 1:**
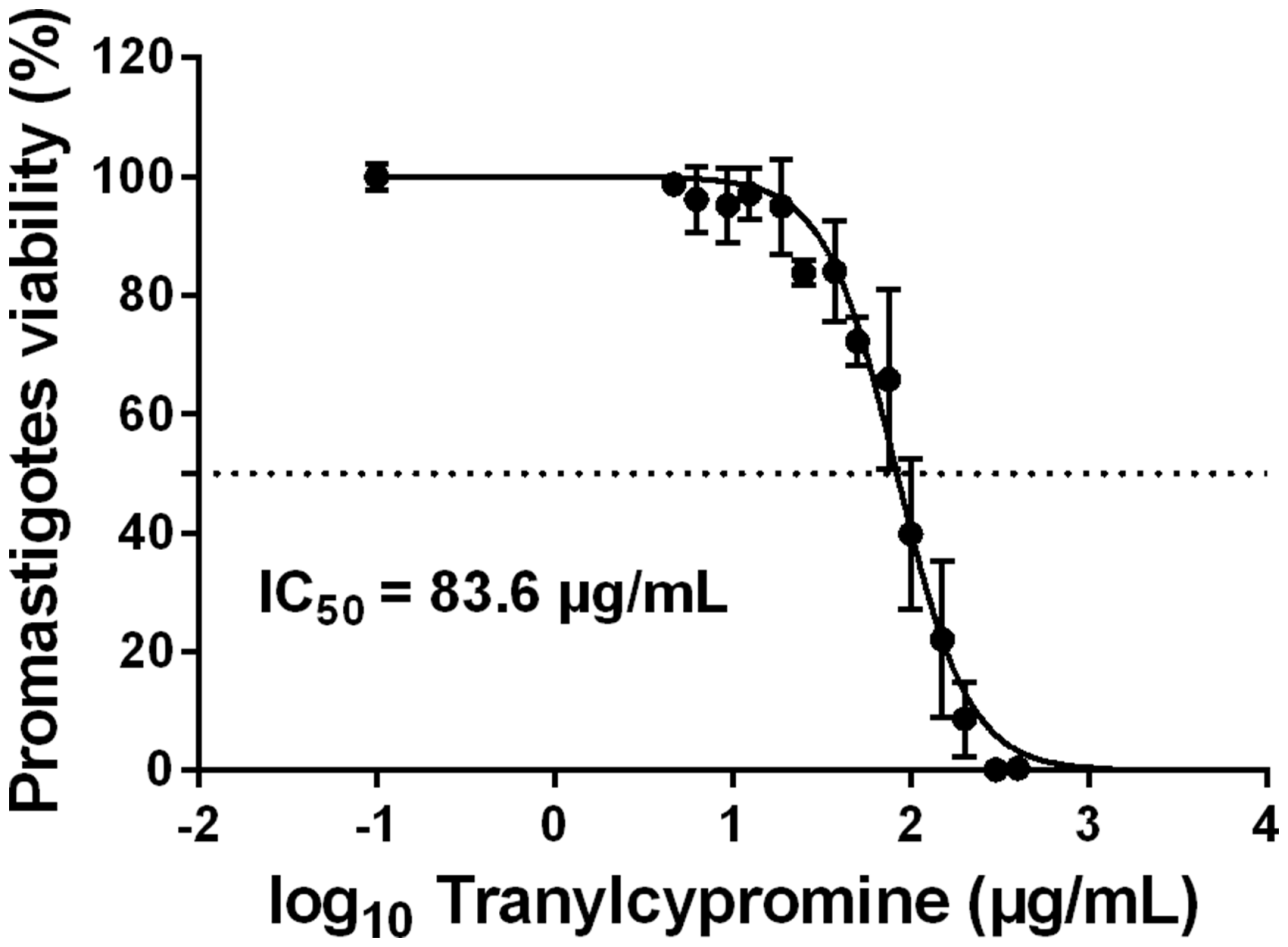
Dose-dependent effect of tranylcypromine on *L. major* (Empa-12) promastigotes. Stationary-phase promastigotes were seeded in 96-well plates at a density of 5 × 10^5^ parasites/well and incubated in the absence or presence of increasing concentrations of tranylcypromine (0, 25, 37.5, 50, 75, 100, 150, 200, 300, and 400 μg/ml). After 24 h of incubation, parasite viability was assessed using an MTT assay. Results are expressed as the percentage of viable promastigotes relative to mock-treated controls and are presented as mean ± standard deviation (SD) of three independent experiments performed in technical duplicates.

To evaluate the effect of Tranylcypromine on promastigotes, 5 × 10^5^ stationary-phase Empa-12 promastigotes were seeded in 96-well plates containing RPMI medium and exposed to increasing concentrations of Tranylcypromine (0 to 400 μg/ml). After 24 h of treatment, the MTT assay showed that the compound induced a moderate decrease in parasite viability at lower concentrations (37.5 to 75 μg/ml), while at 150 μg/ml, viability was reduced by approximately 65%. At a concentration of 300 μg/ml, inhibition exceeded 90%, indicating that Tranylcypromine induced a clear dose-dependent reduction in the viability of *L. major* promastigotes, with an IC_50_ value of 83.6 μg/ml (Figure 1). This dose–response relationship highlighted the potential of Tranylcypromine to interfere with parasite viability *in vitro*. To our knowledge, these results provide the first experimental evidence of the antileishmanial activity of Tranylcypromine, suggesting that this compound may represent a promising candidate for further investigation as a repurposed therapeutic agent against leishmaniases.

### 3.2 Tranylcypromine-induced morphological alterations in *Leishmania* parasites

**Figure 2:**
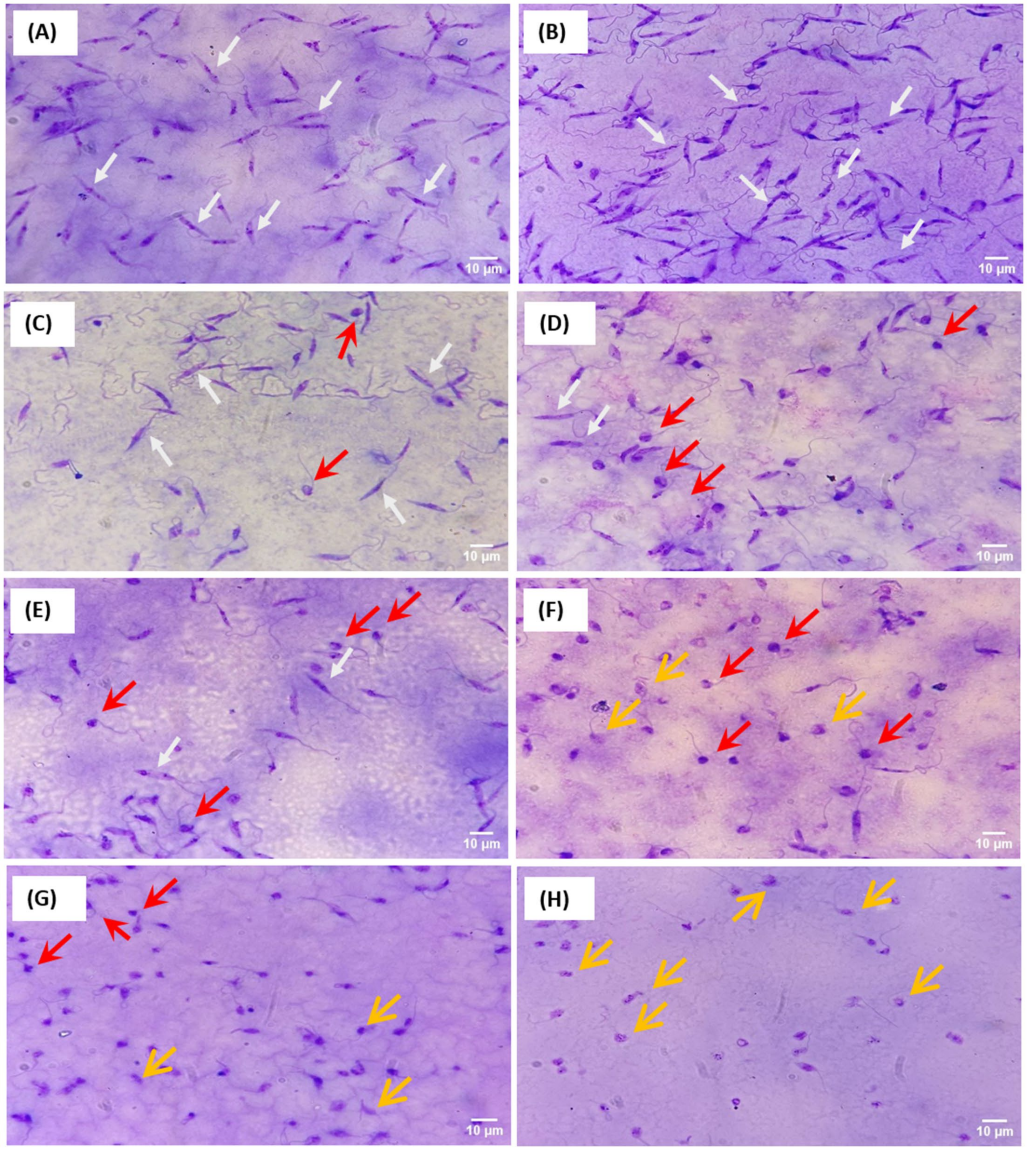
Induced-morphological changes by Tranylcypromine on *L. major* promastigotes. Giemsa-stained promastigotes are shown for mock-treated **(A)** or Tranylcypromine-treated at 25 μg/ml **(B)**, 50 μg/ml **(C)**, 75 μg/ml **(D)**, 100 μg/ml **(E)**, 150 μg/ml **(F)**, 200 μg/ml **(G)**, and 400 μg/ml **(H)**. Slides were examined at 100 × magnification. White arrows indicate intact promastigotes with elongated cell bodies, red arrows highlight apoptotic parasites, and yellow arrows mark necrotic parasites. Scale bars: 10 μm.

Giemsa-stained slides showed that mock-treated promastigotes (Figure 2A) displayed typical elongated and slender cell bodies, characteristic of intact parasites. At 25 μg/ml of Tranylcypromine (Figure 2B), most promastigotes exhibited a normal shape with elongated bodies and long flagella. Interestingly, increasing concentrations of the compound (50, 75, 100, 150 and 200 μg/ml) induced morphological changes that were characterized by cell shrinkage, rounding, and partial flagellar shortening (Figures 2C–G). Starting from the concentration of 150 μg/ml, promastigotes shape was altered and the parasites displayed swollen and irregular cell bodies (Figures 2F–H). The loss of flagella, the disruption of the membrane integrity and the appearance of more rounded shapes were predominant at the highest concentration of Tranylcypromine (400 μg/ml) (Figure 2H). This result indicates that the inhibitory effect induced by Tranylcypromine on the viability of *Leishmania* promastigotes was associated with changes in their normal shape and morphology.

### 3.3 Tranylcypromine induced dose-dependent apoptosis in *Leishmania* promastigotes associated to necrosis at higher concentrations

**Figure 3:**
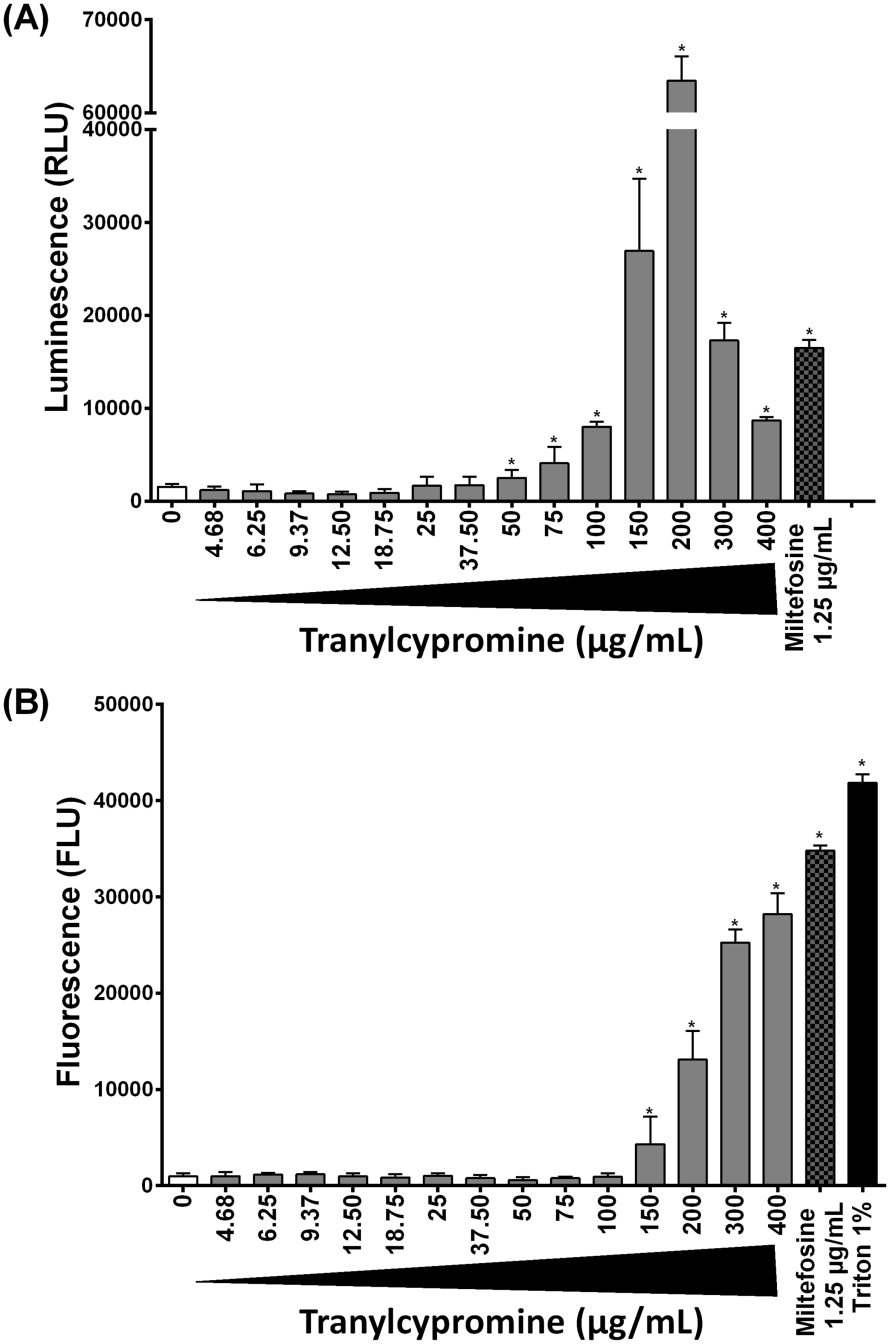
Evaluation of the effect of Tranylcypromine on **(A)** apoptosis and **(B)** necrosis in *L. major* promastigotes (Empa-12). Stationary-phase promastigotes were seeded in 96-well plates at a density of 5 × 10^5^ parasites per well and incubated in the absence or presence of increasing concentrations of Tranylcypromine (0, 4.69, 6.25, 9.38, 18.75, 25, 37.5, 50, 75, 100, 150, 200, 300, and 400 μg/ml). After 24 h of incubation, apoptosis and necrosis were assessed using the RealTime-Glo™ Annexin V Apoptosis and Necrosis Assay (Promega). Reagents were added to measure luminescent signal indicative of apoptosis induction along with fluorescence as a marker of necrosis and membrane integrity. Promastigotes treated with Miltefosine (1.25 μg/ml) were used as a positive control for apoptosis, while Triton X-100 (1%) served as a positive control for necrosis. Readings were performed using a microplate reader. Data are presented as mean ± standard deviation (SD) of three independent experiments conducted in technical duplicate. Statistical significance was determined using Student’s *t*-tests comparing each Tranylcypromine-treated group to the untreated (mock-treated) control *p < 0.05 indicates statistically significant differences.

The progressive morphological alterations induced by Tranylcypromine suggested that this compound could trigger an apoptotic cell death in *Leishmania* promastigotes. To this end, we investigated both the apoptotic and necrotic processes that could occur in response to Tranylcypromine as a mechanism of cell death. Promastigotes were seeded in 96-well plates at 5 × 10^5^ parasites per well and treated with increasing concentrations of Tranylcypromine (0 to 400 μg/ml) for 24 h. Apoptosis and necrosis were measured using the RealTime-Glo™ Annexin V Apoptosis and Necrosis Assay. In this assay, luminescence reflects Annexin V fusion protein binding to exposed phosphatidylserine indicating apoptotic cell death, while fluorescence indicates necrosis and the loss of membrane integrity. At low concentrations (≤ 37.5 μg/ml), Tranylcypromine exhibited a little effect on the viability of promastigotes (Figure 1) that did not coincide with apoptosis nor necrosis induction (Figure 3). Starting from the concentration of 50 μg/ml, the luminescence signal increased in a dose-dependent manner indicating the progressive activation of programmed cell death that was concomitant to inhibition of parasite viability. The apoptotic activity reached a peak at 200 μg/ml (Figure 3A). As expected, the positive control Miltefosine (at 1.25 μg/ml), induced an increase in luminescence, confirming apoptosis induction. However, necrosis was detectable at 150 μg/ml of Tranylcypromine and gradually increased, becoming the predominant form of cell death at concentrations of 300 to 400 μg/ml. It is worth to note that the fluorescence signal at the concentrations of 150–200 μg/ml remained weaker compared to that emitted by the positive controls Miltefosine (1.25 μg/ml) and [Triton X-100 (1%)] as indicative of necrotic cell death and the loss of membrane integrity (Figure 3B).

Taken together, these results indicated that Tranylcypromine is affecting the viability of *L. major* Empa-12 promastigotes through a dual mechanism that was dependent on the tested doses. Apoptosis induction occurred at moderate concentrations of the compound 50–200 μg/ml preceding the shift to necrotic process starting from 150 μg/ml of Tranylcypromine and leading to cytotoxicity and irreversible membrane damage on promastigotes at high concentrations 300–400 μg/ml.

### 3.4 Tranylcypromine induced no cytotoxic effects on host cells at promastigotes’ IC_50_ concentrations

**Figure 4:**
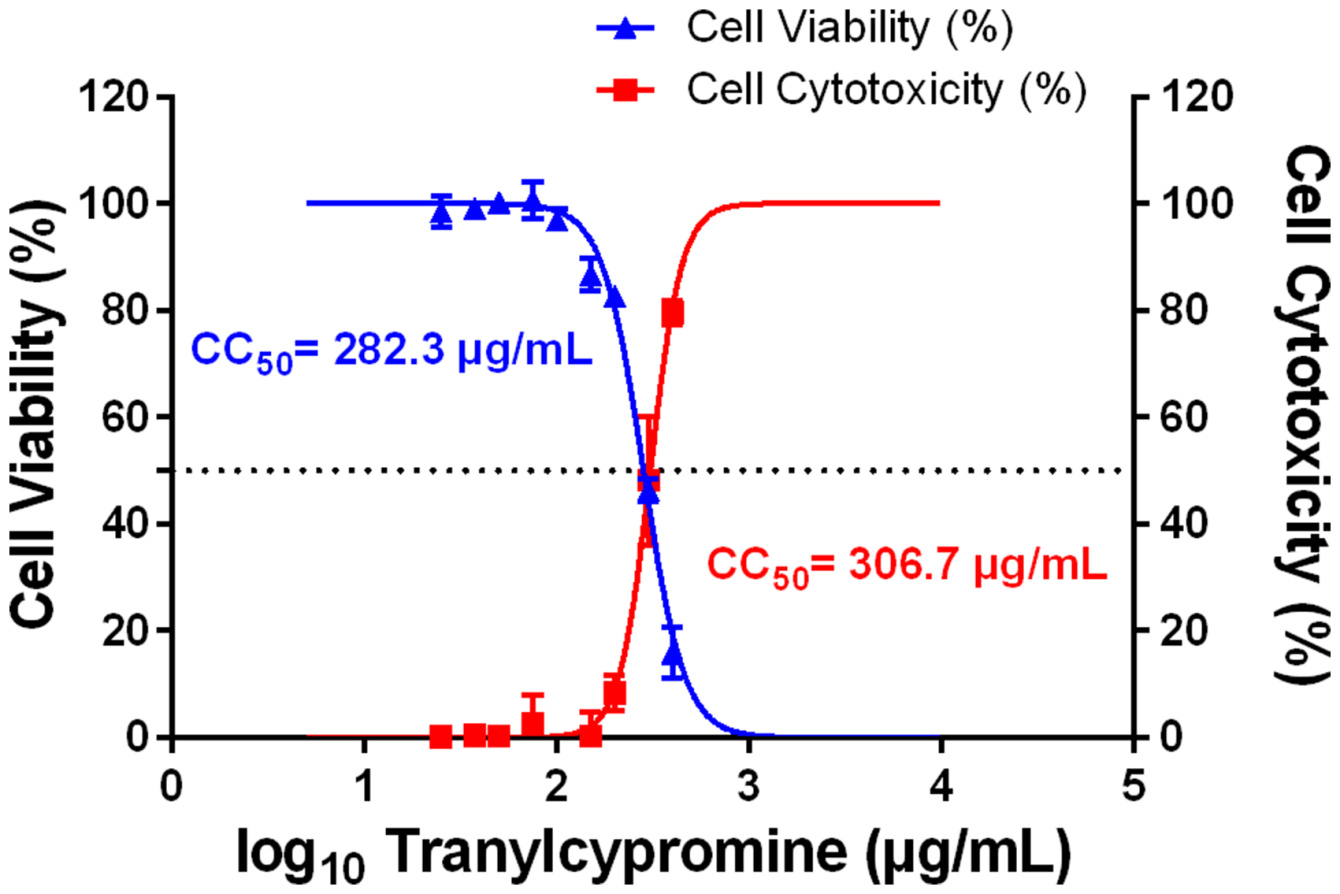
Effect of tranylcypromine on the viability and the lactate dehydrogenase release of THP-1-derived macrophages. THP-1 cells were differentiated into macrophages using PMA (25 ng/ml, 24 h) and then incubated for 24 h with increasing concentrations of tranylcypromine (0, 25, 37.5, 50, 75, 100, 150, 200, 300, and 400 μg/ml). Cell viability was assessed by the MTT assay, and results are expressed as the percentage of viable cells relative to mock-treated controls. Cytotoxicity was evaluated by LDH release, expressed as the percentage of compound-induced enzyme increase compared to the positive control. Data are presented as the mean ± SD of three independent experiments performed in duplicate. Dose–response curves were fitted using nonlinear regression analysis of log-transformed concentrations versus response percentages.

To assess the potential cytotoxic effects of Tranylcypromine on host cells, THP-1-derived macrophages were treated with increasing concentrations of the compound (0 to 400 μg/ml) for 24 h. Cell viability was first evaluated using the MTT assay, while cytotoxicity was investigated also by measuring LDH release in culture supernatants. MTT assay results indicated that Tranylcypromine had no effect on the viability of THP-1-derived macrophages at concentrations up to 100 μg/ml, safely exceeding its IC_50_ against *L. major* (~83 μg/ml). However, a gradual decline in cell viability was observed at higher doses, with noticeable increase in the mortality rate at 300 and 400 μg/ml of the compound. The calculated concentration of Tranylcypromine that reduced cell viability by 50% (CC_50_) was 282.3 μg/ml (Figure 4). Consistently, LDH assay data highlighted the induction of reduced cytotoxicity at doses that did not exceed 150 μg/ml, followed by a marked increase at higher concentrations (300 and 400 μg/ml). The concentration inducing 50% cell cytotoxicity (CC_50_) was estimated to 306.7 μg/ml (Figure 4). Overall, Tranylcypromine did not induce significant cytotoxicity nor viability inhibition at concentrations lower or equal to the calculated IC_50_ value against *Leishmania* promastigotes, thereby supporting its selective anti-parasitic effect.

### 3.5 Tranylcypromine reduced the viability of amastigotes

**Figure 5:**
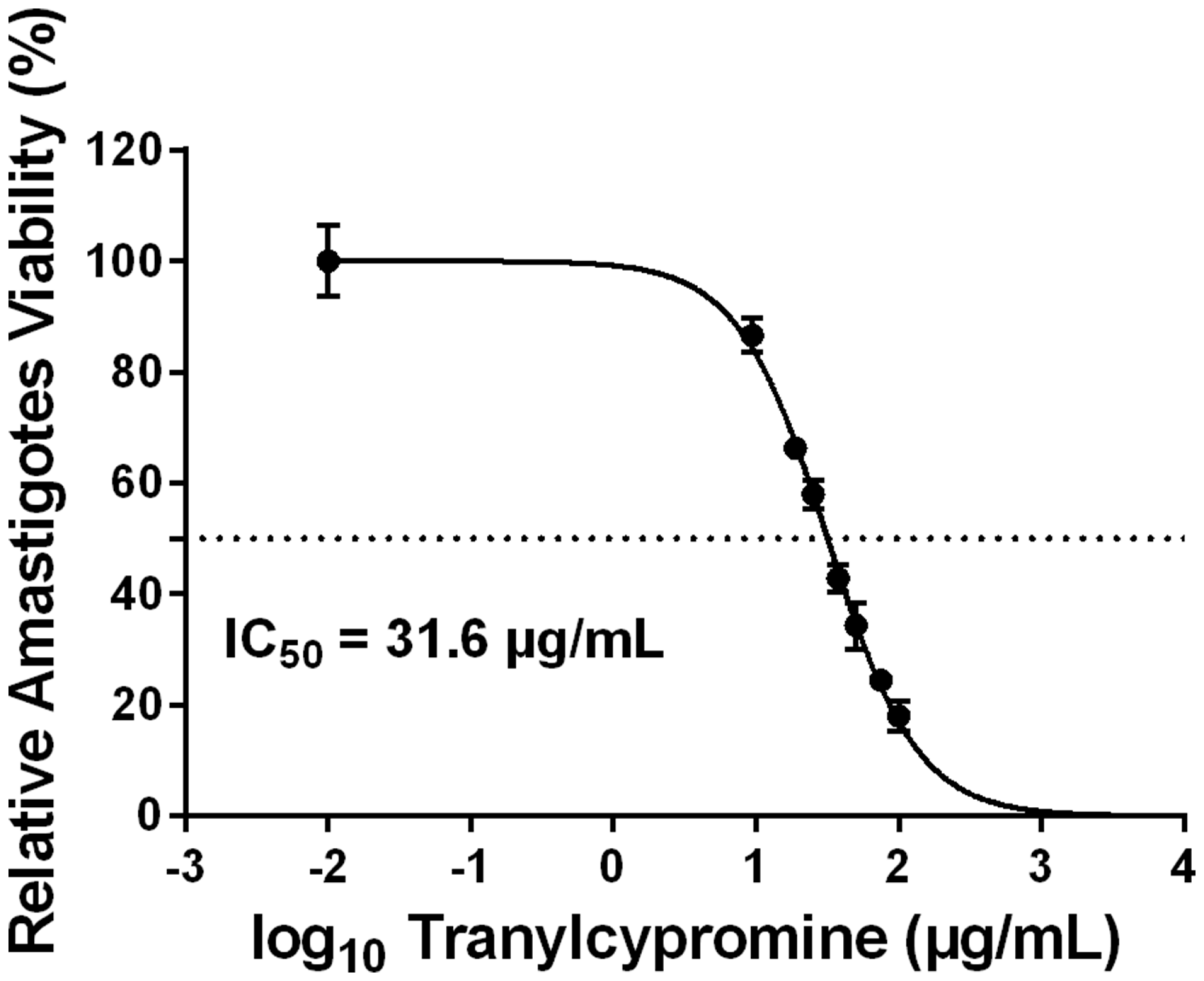
Dose-dependent effect of tranylcypromine on intracellular *Leishmania major* (Empa-12) amastigotes. THP-1-derived macrophages were infected with the *L. major* Empa-12 strain (parasite-to-macrophage ratio 10:1) for 24 h, followed by a 24-h treatment with increasing concentrations of tranylcypromine (0, 9.37, 18.75, 25, 37.5, 50, 75, and 100 μg/ml). After incubation, cells were fixed and stained using the RAL 555 rapid staining kit. The number of intracellular amastigotes per 100 infected macrophages was counted and expressed as a percentage relative to mock-treated controls. Data is presented as the mean ± SD of three independent experiments. Dose–response curves were generated by plotting the logarithm of tranylcypromine concentrations against relative amastigotes viability.

To further evaluate the antileishmanial potential of Tranylcypromine, its effect was tested against intracellular amastigotes of *Leishmania major*. Differentiated THP-1 macrophages were infected with the Empa-12 strain at a parasite-to-cell ratio of 10:1 for 24 h. Following infection, cells were treated for an additional 24 h with increasing concentrations of Tranylcypromine (0 to 100 μg/ml), then fixed and stained using the RAL 555 kit. The intracellular parasite burden was quantified and expressed relative to DMSO-treated controls. We found that Tranylcypromine caused a dose-dependent reduction in the number of intracellular amastigotes with an IC_50_ value of 31.6 μg/ml, approximately 2.6-fold lower than that observed for promastigotes. At 75 μg/ml, the parasite load decreased by nearly 80% (Figure 5). Importantly, no signs of cytotoxicity were detected in host macrophages at the tested concentrations (Figure 5). Overall, these results indicated that Tranylcypromine was active against the intracellular stage of *L. major* while preserving host cell viability and reinforcing its potential as a selective and promising anti-leishmanial candidate.

## 4 Discussion

Drug repurposing has emerged as an effective strategy to accelerate the identification of new therapeutic options while reducing the cost and time associated with conventional drug development. By exploring new indications for compounds with well-established pharmacokinetic and safety profiles, this approach offers a pragmatic route to address unmet medical needs. In this context, antidepressant molecules have been extensively investigated for repurposing. Beyond their primary role in modulating neurotransmission, many antidepressants exhibit a broad range of pharmacological activities, including anti-inflammatory (Patel et al., 2023), immunomodulatory (Nava et al., 2025), antiviral (Wichit et al., 2017), antibacterial (Andersson et al., 2017; Burin and Shah, 2021; Caldara and Marmiroli, 2021), antifungal (Holmes et al., 2012; Moraes and Ferreira-Pereira, 2019; Caldara and Marmiroli, 2021) and even anti-parasitic effects (Andrade-Neto et al., 2018).

Tricyclic antidepressants such as Imipramine, Clomipramine, and Amitriptyline have been reported to reduce parasite viability acting on both promastigote and amastigote forms of *L. donovani* and *L. major* (Zilberstein and Dwyer, 1984; Zilberstein et al., 1990). Imipramine was shown to be effective against intracellular amastigotes that are resistant to antimony, without causing detectable damage to host cells, and oral treatment with this compound reduced parasite burden in a hamster model of visceral leishmaniasis (Mukherjee et al., 2012). Their antileishmanial effects have been linked to multiple mechanisms, including disruption of sterol composition, inhibition of trypanothione reductase, alteration of the proton motive force, and the induction of apoptosis (Zilberstein and Dwyer, 1984; Zilberstein et al., 1990; Benson et al., 1992; Mukherjee et al., 2012, 2014). In addition, Imipramine has been reported to modulate host immune responses by enhancing TNF-α and IFN-γ production while reducing IL-10 and TGF-β expression (Mukherjee et al., 2014). Beyond tricyclic antidepressants, other psychotropic agents have also attracted attention for their activity against *Leishmania*. Another antidepressant, Sertraline, a selective serotonin reuptake inhibitor routinely prescribed for depression and for disorders such as Obsessive-Compulsive Disorder, and generalized anxiety, showed potent activity against *L. donovani*. It showed clear activity on both promastigotes and intracellular amastigotes. In infected BALB/c mice, oral treatment also led to a marked reduction in parasite loads in the spleen and liver. Experiments further indicated that the drug interferes with parasite energy production, as reflected by reduced ATP levels and oxygen consumption in treated promastigotes (Palit and Ali, 2008). Monoamine oxidase inhibitor antidepressants have also been shown to exert antiparasitic activity against both visceral and cutaneous *Leishmania* strains. Among the compounds tested *in vitro*, Phenelzine displayed the strongest activity, whereas *in vivo* studies identified Nialamide as the more effective agent (Evans et al., 1989). Notably, Nialamide also produced beneficial effects when applied topically to cutaneous lesions (Evans et al., 1989). These findings suggest that psychoactive molecules may interfere with conserved metabolic or redox processes essential for parasite survival.

In this context, Tranylcypromine, Monoamine oxidase inhibitor, emerged from our machine learning based repurposing screen as a promising anti-*Leishmania* candidate (Harigua-Souiai et al., 2022). In the present work, we experimentally confirmed this prediction and showed that Tranylcypromine effectively reduces parasite viability. A clear dose-dependent effect was observed against *L. major* promastigotes (IC₅₀ = 83.6 μg/ml), while intracellular amastigotes were even more sensitive (IC₅₀ = 31.6 μg/ml), pointing to efficient penetration and activity within host macrophages. At 75 μg/ml, parasite burden was reduced by approximately 80% while macrophages remained morphologically normal and fully viable, indicating its potential as a therapeutic candidate.

The reference antileishmanial agents such as amphotericin B consistently exhibited its activity against *Leishmania* promastigotes and intracellular amastigotes with IC₅₀ values that did not exceed 1 μg/ml, reflecting its high potency (Pandharkar et al., 2014; Kariyawasam et al., 2019; Oualha et al., 2024). Miltefosine and pentamidine show higher IC₅₀ values against promastigotes (1 to ~50 μg/ml) (Croft et al., 2006; Plourde et al., 2012; Pandharkar et al., 2014; Alshakir and Zghair, 2015). Paromomycin, another established antileishmanial drug, inhibited the viability of *L. tropica* promastigotes at concentrations ranging from 3 to 19 μg/ml (Pandharkar et al., 2014), while another study demonstrate that intracellular amastigotes are sensitive to this drug at a concentration of 40 μg/ml (El-On et al., 2007). It is important to note that, IC₅₀ values reported for antileishmanial compounds vary depending on the *Leishmania* species and the tested strain, as well as experimental parameters such as duration of drug exposure and detection method that complicate direct quantitative comparisons across studies.

Beyond its classical psychiatric applications, Tranylcypromine and its derivatives have shown promise in various repurposing contexts. It was identified as a privileged chemical scaffold targeting multiple enzymes and receptors, including lysine demethylase 1 (LSD1), and cytochrome P450 enzymes (Ulrich et al., 2017; Song et al., 2025). Several LSD1 inhibitors structurally based on Tranylcypromine scaffold (e.g., ORY-2001, ORY-1001, and TAK-418) are currently in clinical trials for cancer therapy, highlighting its emerging potential in oncology (Zheng et al., 2016; Song et al., 2022, 2025; Sheikh et al., 2025). Moreover, Tranylcypromine’s involvement in modulating prostaglandin synthesis and drug metabolism pathways further extended its therapeutic potential. In infectious diseases research, Tranylcypromine derivatives such as MIV-150 have progressed to clinical evaluation as reverse transcriptase inhibitors for HIV-1 (Li et al., 2022; Song et al., 2025). Overall, Tranylcypromine’s unique molecular architecture and multifunctional pharmacological profile have positioned it as a privileged scaffold for novel therapeutic agents. We herein, showed through mechanistic assays that Tranylcypromine induced apoptosis in promastigotes at moderate concentrations reaching the highest apoptotic response at 200 μg/ml followed by necrosis at increasing doses. This dual profile was consistent with observations for other antileishmanial compounds (Zahir et al., 2015; Silva-Silva et al., 2022), suggesting the involvement of oxidative stress or mitochondrial dysfunction in the induction of cell death. This study serves as the first experimental validation of Tranylcypromine as an antileishmanial candidate, and future studies are warranted to elucidate its mechanism action of in *Leishmania.* In particular, future additional experiments such as reactive oxygen species (ROS) measurements, mitochondrial membrane potential analyses, or caspase-like activity assays would be valuable to deeper investigate the cellular and molecular processes associated to Tranylcypromine activity.

While Tranylcypromine is a monoamine oxidase inhibitor in humans, there is currently no evidence that *Leishmania* parasites express classical monoamine oxidases. Therefore, in this study, we cannot attribute its antileishmanial activity to MAO inhibition nor exclude that other mechanisms could also be implicated. Consequently, the molecular target(s) of Tranylcypromine in the *Leishmania* parasite remain to be investigated. Several drugs such as Miltefosine, Amphotericin B, Pentavalent antimonials, and Paromomycin exhibited anti-parasitic activity and are currently used to treat leishmaniases even if their molecular targets in the parasite are still not completely defined (Croft et al., 2006; Seifert, 2011; Zhang et al., 2025). Many of these compounds were originally developed for other applications and later repurposed for leishmaniases (Andrade-Neto et al., 2018; Lye et al., 2024). This illustrates a common situation in antileishmanial therapy where effective drugs may act through mechanisms that are not yet fully understood, highlighting the importance of mechanistic studies to uncover their targets and inform the development of future therapies.

Noticeably, Tranylcypromine exhibited low cytotoxicity toward THP-1-derived macrophages (CC_50_ > 280 μg/ml), resulting in a satisfactory selectivity index of ~9. The absence of morphological alterations or LDH release at therapeutically relevant concentrations further supported its selective actions. This favourable safety profile, its known oral administration, provides a significant advantage over standard antileishmanial therapies such as pentavalent antimonials or amphotericin B, which require parenteral delivery and are associated with substantial toxicity. The combination of low toxicity, oral bioavailability, and selective antiparasitic activity also supports potential use in combination therapies, which could enhance antiparasitic effects, reduce doses of more toxic drugs, and limit the emergence of resistance. In fact, the pharmacokinetics of Tranylcypromine are well characterized, with rapid and efficient absorption following oral administration and peak plasma concentrations typically reached within 1 to 3 h. It undergoes extensive hepatic metabolism and exhibits a relatively short half-life of approximately 1.5 to 3.2 h (Ulrich et al., 2017). Its safety profile has been established over decades of clinical use, providing a favourable therapeutic window essential for repurposing efforts (Ulrich et al., 2017; Song et al., 2025).

Overall, our findings have positioned Tranylcypromine as a promising starting point for further optimization, reinforcing the broader potential of psychoactive drugs as a source of novel candidates for neglected diseases therapies. Nonetheless, continued investigations shall determine whether Tranylcypromine or improved derivatives can advance toward preclinical development, while observing special attention to remaining outside the therapeutic doses inferring psychiatric effects.

The present research highlighted the value of AI-driven drug repurposing for uncovering new therapeutic opportunities against infectious diseases, such as Leishmaniases. Tranylcypromine appeared as a promising molecule, showing selective activity against the parasite with limited toxicity to host cells, while benefiting from oral administration and an extensively documented clinical safety profile. These results provided a strong rationale for advancing this compound in the antileishmanial drug discovery pipeline. Nonetheless, future work should therefore focus on validating the antiparasitic effect *in vivo*, characterizing its molecular mechanisms, assessing pharmacokinetic/pharmacodynamic compatibility with antileishmanial therapy, and exploring synergistic combinations aimed at reducing toxicity and limiting drug resistance.

## Data Availability

The original contributions presented in the study are included in the article/supplementary material, further inquiries can be directed to the corresponding author.
